# Pre-existing atrial fibrillation and risk of arterial thromboembolism and death in intensive care unit patients: a population-based cohort study

**DOI:** 10.1186/s13054-015-1007-5

**Published:** 2015-08-19

**Authors:** Jacob Gamst, Christian Fynbo Christiansen, Bodil Steen Rasmussen, Lars Hvilsted Rasmussen, Reimar Wernich Thomsen

**Affiliations:** Department of Clinical Epidemiology, Institute of Clinical Medicine, Aarhus University Hospital, Olof Palmes Allé 43-45, DK-8200 Aarhus N, Denmark; Department of Cardiology, Aalborg University Hospital, Hobrovej 18-22, DK-9000 Aalborg, Denmark; Department of Anaesthesia and Intensive Care Medicine, Aalborg University Hospital, Hobrovej 18-22, DK-9000 Aalborg, Denmark; Aalborg Atrial Fibrillation Study Group, Aalborg University Hospital Science and Innovation Centre, Søndre Skovvej 15, DK-9000 Aalborg, Denmark; Faculty of Medicine, Aalborg University, Niels Jernes Vej 10, DK-9220 Aalborg Øst, Denmark

## Abstract

**Introduction:**

Pre-existing atrial fibrillation (AF) may worsen prognosis in patients admitted to the intensive care unit (ICU).

**Methods:**

In a cohort study (2005–2011) including all patients with first-time ICU admissions in Denmark (n=57,110), we compared patients with and without pre-existing AF and estimated absolute risks and relative risks (RRs) of arterial thromboembolism and death within 30 days and 365 days following admission, using Kaplan-Meier methods and multivariate regression analyses. We analysed the prognostic impact of AF within strata of patient age, sex, coexisting cardiac diseases, and ICU therapies.

**Results:**

Among ICU patients, 5065 (9 %) had pre-existing AF. Compared with patients without AF, those with AF were older (median age 75 vs. 62 years) and had more comorbidity. The risk of arterial thromboembolism was 2.8 % in patients with AF and 2.0 % in non-AF patients at 30 days, and 4.3 % and 2.9 %, respectively, at 365 days. Corresponding RRs were 1.41 crude and 1.14 (95 % confidence interval [CI] 0.93–1.40) adjusted at 30 days, and 1.50 crude and 1.20 (95 % CI 1.02–1.41) adjusted at 365 days. Thirty-day mortality was 27 % in patients with pre-existing AF and 16 % in non-AF patients (crude RR 1.67, adjusted RR 1.04, 95 % CI 0.99–1.10). Corresponding mortality estimates at 365 days were 40.9 % and 25.4 %, respectively (crude RR 1.61, adjusted RR 1.03, 95 % CI 1.00–1.07). In stratified analyses, pre-existing AF increased mortality in ICU patients aged <55 years (adjusted RR at 30 days 1.73, 95 % CI 1.29–2.32; adjusted RR at 365 days 1.34, 95 % CI 1.06–1.69) and in ICU patients treated with mechanical ventilation (adjusted RR at 30 days 1.12, 95 % CI 1.05–1.20, adjusted RR at 365 days 1.09, 95 % CI: 1.04–1.15). Analyses stratified by sex and coexisting cardiac diseases yielded adjusted RRs close to 1.

**Conclusions:**

In ICU patients, pre-existing AF was associated with modestly increased risk of arterial thromboembolism when adjusted for the substantially higher age and comorbidity levels in patients with AF, whereas there was no overall association with mortality. In ICU patients aged <55 years and in those treated with mechanical ventilation, AF predicted increased mortality.

**Electronic supplementary material:**

The online version of this article (doi:10.1186/s13054-015-1007-5) contains supplementary material, which is available to authorized users.

## Introduction

The overall burden of pre-existing chronic diseases is an important determinant of prognosis for patients admitted to the intensive care unit (ICU) [1, 2]. Chronic diseases are reportedly more prevalent in ICU patients than in the general population, and with the ageing of the population, the prevalence of chronic diseases is expected to increase in coming years [1, 3]. Despite this, the prognostic influence of individual coexisting diseases in ICU patients remains poorly investigated.

Atrial fibrillation (AF) is the most common heart rhythm disorder, affecting 1.0–1.8 % of the adult population in western countries [4, 5]. In the general population, AF has been associated with substantially increased risk of mortality, stroke, and heart failure [6–8]. In patients admitted to the ICU, new-onset AF has been linked to increased risk of death [9, 10]. To date, evidence on how, and to what extent, pre-existing AF affects the prognosis for ICU patients is sparse. Pre-existing AF could potentially have serious consequences in critically ill patients. The ability to compensate for haemodynamic alterations during critical illness may be reduced in patients with AF, and the inflammatory response could increase the risk of thrombosis formation during ICU stay [11]. These possible complications of AF are likely more pronounced with increased severity of illness during the ICU admission. Also, on the one hand, as the prognostic impact of AF increases with advancing age, it is possible that the effect of AF is influenced by patient age [12, 13]. On the other hand, the potentially detrimental effects of AF may be modified by effective medications prescribed for AF, such as anticoagulants and heart rhythm–modulating drugs.

The prevalence of AF is expected to increase considerably in the coming years with the increasing ageing population and prevalence of comorbidities, and elderly patients with comorbidities are increasingly admitted to the ICU [5, 14, 15]. It is therefore of major importance to elucidate the prognostic impact of AF in ICU patients of different ages and in those with and without comorbidities. Thus, we conducted a large, population-based cohort study to examine the effect of pre-existing AF and associated pharmacotherapy on the risk of arterial thromboembolism (ATE) and death following ICU admission. We also sought to investigate whether any prognostic effect of AF differed according to patient age, sex, and comorbidity and with regard to the reason for and treatment during ICU admission.

## Methods

This nationwide cohort study was conducted in Denmark, a nation with approximately 5.6 million inhabitants. The study was based on prospectively collected data from medical and administrative databases. Each Danish resident is assigned a unique personal identifying number, which enables unambiguous crosslinking of public registries for research purposes. The Danish national health care system offers unrestricted, tax-funded hospital and primary care to all its citizens.

### Identification of the cohort and ICU data

All adult patients (age 15+ years) with a first-time ICU admission in Denmark between January 2005 and December 2011 were included in the cohort. We used the Danish Intensive Care Database (DID) to identify the cohort members. The DID maintains data on all ICU admissions in Denmark from 2005 onward [16]. From the DID, we also retrieved data on treatment with mechanical ventilation, non-invasive ventilation, inotropes and/or vasopressors, and renal replacement therapy during the index ICU admission. To differentiate between ICU admissions related to recent surgery and medical ICU admissions, we obtained data from the Danish National Patient Register (DNPR) on surgical procedures performed within 7 days preceding the ICU admission. The DNPR includes all patients admitted to Danish hospitals since 1977 and holds dates of admission and discharge, surgical procedure codes, and diagnostic codes [17]. We assessed surgical procedures as any type of surgery and specified according to cardiac, abdominal, orthopaedic, or vascular surgery.

### Data on atrial fibrillation

We used the DNPR to identify cohort members with previously diagnosed AF. We considered diagnoses of AF assigned during either in-hospital admission or at a hospital specialist outpatient clinic visit within 5 years before the index ICU admission. Because AF and atrial flutter share the same code in the DNPR, we were not able to distinguish between these arrhythmias. However, it has been demonstrated that, among patients coded with AF or atrial flutter in the DNPR, 92–95 % have AF [18, 19]. Data on pre-admission treatment for AF was acquired from the Danish Health Service Prescription Registry, which holds data on all prescriptions dispensed from all Danish pharmacies [20]. We noted prescriptions for the most frequently used medications for AF (vitamin K antagonists, aspirin, beta-blockers, non-dihydropyridine calcium-channel blockers, amiodarone, and digoxin) [21]. We also included data on statins, which are both frequently prescribed to individuals with cardiovascular disease and associated with favourable prognosis in ICU patients [22].

### Data on potential confounders

To control our estimates for confounding by coexisting diseases, we retrieved data from the DNPR on diagnoses from previous hospital contacts (in-hospital admission, outpatient clinic, or emergency department) at any time before ICU admission. For the thromboembolism analyses, we identified the risk factors included in the CHA_2_DS_2_-VASc score (i.e., congestive heart failure, hypertension, age, diabetes, prior stroke/transient ischaemic attack/thromboembolism, vascular disease, and sex category), which has been validated for prediction of stroke in patients with AF [13, 23]. For the mortality analyses, we included data on the 19 disease categories in the Charlson comorbidity index, which have been validated for prediction of hospital and ICU mortality [24–26]. For the mortality analyses, we further included diagnoses of valvular heart disease, angina pectoris and chronic ischaemic heart disease, hypertension, alcoholism, and obesity. To address any imbalances in frailty or health awareness not accounted for by previously diagnosed diseases, we additionally retrieved data from the National Health Service Register, which includes consultations and procedures provided by primary health care providers [27], on preventive consultations, social medicine–related consultations, application for reimbursement due to chronic or terminal illness, conversational therapy, and influenza vaccinations by the general practitioner (GP) within 1 year preceding the index ICU admission.

### Outcomes

The outcomes were ATE and death. We evaluated both outcomes at 30 days and 365 days following the ICU admission date. We defined ATE as a diagnosis of non-haemorrhagic stroke or thrombosis or embolism in arteries of the extremities, the mesenteric arteries, or unspecified arteries recorded in the DNPR. The Civil Registration System provided data on vital status of the cohort members. This database, which is updated daily, encompasses all Danish residents since 1968 and holds information on exact dates of birth, death, and migration [28].

### Statistical analyses

Follow-up began at ICU admission and continued until 1 January 2013. We computed the cumulative risk of ATE in patients with and without pre-existing AF, accounting for the competing risk of death in the analyses [29]. To further compare the risk of ATE in patients with AF with that in non-AF patients, we calculated cumulative risk ratios (CRRs), both crude and adjusted for prevalence of the risk factors in the CHA_2_DS_2_-VASc score. Both cumulative risks and CRRs were computed by analysing pseudo values for each observation in a generalized linear model [30, 31]. Within each of the cohorts of patients with and without pre-existing AF, we further investigated the effect of pre-admission use of vitamin K antagonists and aspirin on the risk of ATE by comparing users with non-users. Because the DNPR records all diagnoses assigned during an admission to the same date, some episodes of ATE may have been present before the patient was transferred to the ICU if the ATE and ICU admission occurred during the same hospital admission. In sensitivity analyses, we therefore estimated the cumulative risk and CRR of ATE at 30 days and 365 days following ICU admission, considering only ATE diagnosed at a later hospital admission than the hospital admission during which the patient was treated at the ICU.

Using the Kaplan-Meier method, we estimated mortality risks in patients with pre-existing AF and non-AF patients and compared these by computing relative risks (RRs) through generalized linear regression analyses of pseudo observations [30, 32]. The RRs were presented as crude estimates and estimates adjusted for age, sex, comorbidities (the 19 disease categories in the Charlson comorbidity index, valvular heart disease, angina pectoris and chronic ischaemic heart disease, hypertension, alcoholism, and obesity), and frailty markers as defined by GP services (preventive consultations, social medicine–related consultations, reimbursement due to chronic or terminal illness, conversational therapy, and influenza vaccinations). To investigate whether any effect of AF was different across subgroups of ICU patients, we divided the cohort into three age groups of equal size (15–54, 55–70, and 71–103 years) and repeated the analyses within each age group. Further, we conducted the analyses within strata defined by sex, previously diagnosed myocardial infarction and congestive heart failure, use of mechanical ventilation, non-invasive ventilation, inotropes and vasopressor agents, and renal replacement therapy during the ICU admission, as well as type of surgery performed in relation to the ICU admission. To examine to effect of pre-admission treatment for AF, we assessed absolute risks and RRs of death in users of statins, vitamin K antagonists, aspirin, beta-blockers, non-dihydropyridine calcium-channel blockers, amiodarone, and digoxin as compared with non-users in patients with and without AF separately.

Statistical analyses were performed using Stata 11.2 software (StataCorp, College Station, TX, USA), including the user-written st0202 package.

### Ethics

The study was approved by the Danish Data Protection Agency (record number 2009-41-3987). Informed consent was not required for this registry study according to Danish legislation.

## Results

The study included 57,110 ICU patients, of whom 5065 (9 %) had pre-existing AF (Table 1). Follow-up was incomplete for 172 individuals (169 emigrated and 3 disappeared). The participants’ characteristics are presented in Table 1.

**Table 1:** Characteristics of 57,110 ICU patients with and without pre-existing atrial fibrillation

	AF, n (%)	No AF, n (%)	Total, n (%)
Participants	5065 (8.9)	52,045 (91.1)	57,110 (100.0)
Male	3162 (62.4)	28,851 (55.4)	32,013 (56.1)
Age, yr, median (IQR)	75 (67–81)	62 (47–73)	64 (49–75)
Age groups			
15–55 yr	257 (5.1)	18,247 (35.1)	18,504 (32.4)
55–71 yr	1534 (30.3)	17,212 (33.1)	18,746 (32.8)
71–103 yr	3274 (64.6)	16,586 (31.9)	19,860 (34.8)
Comorbid conditions			
Prior myocardial infarction	1056 (20.1)	5082 (9.8)	6138 (10.8)
Congestive heart failure	1714 (33.8)	3213 (6.2)	4927 (8.6)
Peripheral artery disease	824 (16.3)	4874 (9.4)	5698 (10.0)
Valvular heart disease	1215 (24.0)	3756 (7.2)	4971 (8.7)
Hypertension	2457 (48.5)	10,997 (21.1)	13,454 (23.6)
Angina pectoris	1506 (29.7)	7690 (14.8)	9196 (16.1)
Chronic ischaemic heart disease	1724 (34.0)	6292 (12.1)	8016 (14.0)
Cerebrovascular disease	1217 (24.0)	6386 (12.3)	7603 (13.3)
Hemiplegia	35 (0.7)	420 (0.8)	455 (0.8)
Transient cerebral ischaemic attack	359 (7.1)	1932 (3.7)	2291 (4.0)
Dementia	109 (2.2)	542 (1.0)	651 (1.1)
Chronic pulmonary disease	1336 (26.4)	7769 (14.9)	9105 (16.0)
Connective tissue disease	360 (7.1)	2294 (4.4)	2654 (4.7)
Moderate to severe liver disease	43 (0.8)	614 (1.2)	657 (1.1)
Mild liver disease	117 (2.3)	1597 (3.1)	1714 (3.0)
Diabetes	830 (16.4)	4748 (9.1)	5578 (9.8)
Diabetes mellitus types 1 and 2 with end-organ damage	522 (10.3)	2673 (5.1)	3195 (5.6)
Moderate to severe renal disease	518 (10.2)	2110 (4.1)	2628 (4.5)
Any tumour	967 (19.1)	8750 (16.8)	9717 (17.0)
Leukaemia	27 (0.5)	330 (0.63)	357 (0.6)
Lymphoma	90 (1.8)	651 (1.3)	741 (1.3)
Metastatic solid tumour	149 (2.9)	1604 (3.1)	1753 (3.1)
AIDS	1 (0.0)	64 (0.1)	65 (0.1)
CHA_2_DS_2_-VASc score			
0	226 (4.5)	11,608 (22.3)	11,834 (20.7)
1	575 (11.4)	15,704 (30.2)	16,279 (28.5)
2+	4264 (84.1)	24,733 (47.5)	28,997 (50.8)
Lifestyle-related diagnoses			
Alcoholism	221 (4.4)	3155 (6.1)	3376 (5.9)
Obesity	491 (9.7)	2908 (5.6)	3399 (6.0)
Consultations and services at the GP^a^			
Seasonal influenza vaccination	2557 (50.1)	13,510 (26.0)	16,067 (28.1)
Preventive consultation	305 (6.0)	2894 (5.6)	3199 (5.6)
Social medicine–related consultation	646 (12.8)	3773 (7.3)	4419 (7.7)
Conversational therapy	70 (1.4)	1646 (3.2)	1716 (3.0)
Reimbursement for chronic illness	73 (1.4)	621 (1.2)	694 (1.2)
Reimbursement for terminal illness	9 (0.2)	70 (0.1)	79 (0.1)
Pre-admission cardiovascular drug use^b^			
Statins	1716 (33.9)	11,031 (21.2)	12,747 (22.3)
Aspirin	1988 (39.3)	10,708 (20.6)	12,696 (22.2)
Vitamin K antagonists	2500 (49.4)	1339 (2.6)	3839 (6.7)
Beta-blockers	2742 (54.1)	9254 (17.8)	11,996 (21.0)
Non-dihydropyridine calcium-channel blockers	360 (7.1)	901 (1.7)	1261 (2.2)
Digoxin	1668 (33.0)	779 (1.5)	2447 (4.3)
Amiodarone	363 (7.2)	115 (0.2)	478 (0.8)
ICU therapy			
Mechanical ventilation	2114 (41.7)	18,410 (35.4)	20,524 (36.0)
Non-invasive ventilation	550 (11.0)	3772 (7.3)	4322 (7.6)
Renal replacement therapy	254 (5.0)	1432 (2.8)	1686 (3.0)
Treatment with inotropes or vasopressors	2057 (40.6)	14,987 (28.8)	17,044 (29.8)
Surgery performed 0–7 days before ICU admission			
Any surgical procedure	3039 (60.0)	32,160 (61.8)	35,199 (61.6)
Cardiac surgery	1038 (20.5)	6669 (12.8)	7707 (13.5)
Vascular surgery	179 (3.5)	2764 (5.3)	2943 (5.2)
Abdominal surgery	899 (17.8)	9608 (18.5)	10,507 (18.4)
Orthopaedic surgery	237 (4,7)	2594 (5.0)	2831 (5.0)
Non-surgical ICU admission	2026 (40.0)	19,885 (38.2)	21,911 (38.4)

*AF* atrial fibrillation, *IQR* interquartile range, *GP* general practitioner

^a^Within 1 year preceding ICU admission

^b^Defined as reimbursed prescription within 100 days preceding the ICU admission

### Risk of arterial thromboembolism

At 30 days following ICU admission, the cumulative risk of ATE was 2.8 % (95 % CI 2.4–3.3 %) in patients with pre-existing AF and 2.0 % (95 % CI 1.9–2.2 %) in patients without AF (Table 2). The corresponding CRR adjusted for prevalence of the risk factors included in the CHA_2_DS_2_-VASc score was 1.14 (95 % CI 0.93–1.40). Risks of ATE at 365 days were 4.3 % (95 % CI 3.7–4.4 %) and 2.9 % (95 % CI 2.7–3.0 %) for patients with and without pre-existing AF, respectively, with a confounder-adjusted CRR of 1.20 (95 % CI 1.02–1.41). Considering only ATE diagnosed at a later hospital admission than the hospital admission during which the patient was treated in the ICU, the 30-day risk of ATE was 0.3 % (95 % CI 0.1–0.5 %) in patients with pre-existing AF and 0.2 % (95 % CI 0.2–0.3 %) in patients without AF, with an adjusted CRR of 0.94 (95 % CI 0.35–2.52), and 365-day risks were 1.7 % (95 % CI 1.3–2.0 %) and 1.0 % (95 % CI 0.9–1.1 %) for patients with and without pre-existing AF, respectively, with an adjusted CRR of 1.24 (95 % CI 0.92–1.67) (Additional file 1: Table S1).

**Table 2:** Risk of arterial thromboembolism at 30 days and 365 days following ICU admission in patients with and without atrial fibrillation and by pre-admission use of aspirin and vitamin K antagonists

	30-day ATE risk		365-day ATE risk	
	AF	No AF	AF	No AF
Overall				
Cumulative risk, %	2.8 (2.4–3.3)	2.0 (1.9–2.2)	4.3 (3.7–4.9)	2.9 (2.7–3.0)
Cumulative risk ratio				
Crude	1.41 (1.19–1.67)	1 (ref)	1.50 (1.31–1.73)	1 (ref)
Adjusted^a^	1.14 (0.93–1.40)	1 (ref)	1.20 (1.02–1.41)	1 (ref)
Aspirin users				
Cumulative risk, %				
Non-users	2.5 (2.0–3.1)	2.0 (1.8–2.1)	3.7 (3.1–4.4)	2.7 (2.5–2.8)
Users	3.4 (2.6–4.2)	2.4 (2.1–2.7)	5.1 (4.1–6.1)	3.5 (3.2–3.9)
Cumulative risk ratio				
Non-users	1 (ref)	1 (ref)	1 (ref)	1 (ref)
Users, crude	1.35 (0.98–1.86)	1.21 (1.05–1.40)	1.36 (1.05–1.77)	1.31 (1.17–1.47)
Users, adjusted^a^	1.05 (0.64–1.75)	0.78 (0.65–0.93)	1.13 (0.81–1.59)	0.81 (0.70–0.95)
Vitamin K antagonist users				
Cumulative risk, %				
Non-users	3.4 (2.7–4.1)	2.0 (1.9–2.2)	4.9 (4.1–5.7)	2.9 (2.7–3.0)
Users	2.4 (1.8–3.0)	1.9 (1.2–2.7)	3.7 (2.9–4.4)	3.1 (2.2–4.0)
Cumulative risk ratio				
Non-users	1 (ref)	1 (ref)	1 (ref)	1 (ref)
Users, crude	0.70 (0.50–0.96)	0.95 (0.65–1.39)	0.75 (0.58–0.97)	1.07 (0.79–1.46)
Users, adjusted^a^	0.57 (0.26–1.25)	0.71 (0.46 -1.12)	0.76 (0.50–1.16)	0.82 (0.58–1.16)

*AF* atrial fibrillation, ATE arterial thromboembolism

95 % confidence intervals are given in parentheses

^a^Adjusted for the risk factors included in the CHA_2_DS_2_-VASc score (i.e., congestive heart failure, hypertension, age, diabetes, prior stroke, vascular diseases, and sex category)

In patients with AF who were pre-admission users of aspirin, the adjusted CRR of ATE was 1.05 (95 % CI 0.64–1.75) at 30 days and 1.20 (95 % CI 1.02–1.41) at 365 days following ICU admission compared with patients with AF who were non-users of aspirin. Among AF patients, pre-admission use of vitamin K antagonists was associated with adjusted CRRs of 0.57 (95 % CI 0.26–1.25) at 30 days and 0.76 (95 % CI 0.50–1.16) at 365 days following admission.

### Mortality

The 30-day mortality was 26.8 % (95 % CI 25.6 %–28.0 %) in patients with pre-existing AF and 16.0 % (95 % CI 15.7 %–16.4 %) in patients without AF (Table 3). The confounder-adjusted RR for 30-day mortality of patients with and without pre-existing AF was 1.04 (95 % CI 0.99–1.10). In the youngest third of the cohort (ages 15–55 years), increased mortality was observed with pre-existing AF (adjusted RR 1.73, 95 % CI 1.29–2.32), whereas pre-existing AF did not increase mortality in the two older age groups (55–71 years and 71–105 years) after controlling for confounders (adjusted RR 1.09, 95 % CI 0.96–1.24; adjusted RR 1.04, 95 % CI 0.98–1.10). In those who received mechanical ventilation during the ICU admission, AF was associated with increased 30-day mortality even after confounder adjustment (adjusted RR 1.12, 95 % CI 1.05–1.20).

**Table 3:** Mortality at 30 days and 365 days following admission in 57,110 adult ICU patients with and without atrial fibrillation

	AF, %	No AF, %	Crude RR^a^	Adjusted RR^a,b^
30-day mortality				
Overall	26.8 (25.6–28.0)	16.0 (15.7–16.4)	1.67 (1.59–1.76)	1.04 (0.99–1.10)
Age				
15–54 yr	13.6 (10.0–18.5)	6.1 (5.7–6.4)	2.25 (1.64–3.08)	1.73 (1.29–2.32)
55–70 yr	16.7 (14.9–18.7)	14.6 (14.1–15.1)	1.14 (1.02–1.29)	1.09 (0.96–1.24)
71–105 yr	32.6 (31.0–34.2)	28.5 (27.8–29.2)	1.14 (1.08–1.21)	1.04 (0.98–1.10)
Sex				
Female	30.0 (28.0–32.1)	16.7 (16.2–17.2)	1.80 (1.67–1.93)	1.05 (0.97–1.14)
Male	24.9 (23.4–26.4)	15.5 (15.1–16.0)	1.60 (1.50–1.71)	1.04 (0.97–1.12)
Prior myocardial infarction				
Not present	26.2 (24.9–27.6)	15.6 (15.3–16.0)	1.68 (1.59–1.77)	1.04 (0.98–1.11)
Present	29.1 (26.4–31.9)	20.0 (18.9–21.1)	1.46 (1.31–1.63)	1.06 (0.93–1.19)
Congestive heart failure				
Not present	24.2 (22.8–25.7)	15.3 (15.0–15.6)	1.58 (1.49–1.69)	1.04 (0.97–1.10)
Present	31.9 (29.7–34.1)	27.7 (26.2–29.3)	1.15 (1.05–1.26)	1.08 (0.99–1.18)
Mechanical ventilation				
No	24.9 (24.3–25.5)	11.2 (10.9–11.5)	2.10 (1.96–2.26)	1.00 (0.93–1.08)
Yes	31.3 (29.4–33.3)	23.6 (22.1–25.1)	1.26 (1.18–1.35)	1.12 (1.05–1.20)
Renal replacement therapy				
No	25.9 (24.7–27.1)	15.3 (15.0–15.6)	1.69 (1.60–1.78)	1.04 (0.98–1.09)
Yes	44.1 (38.2–50.4)	41.7 (39.2–44.3)	1.06 (0.91–1.23)	1.04 (0.88–1.22)
Treatment with inotropes or vasopressors				
No	24.3 (22.8–25.9)	12.1 (11.7–12.4)	2.02 (1.88–2.16)	1.02 (0.94–1.09)
Yes	30.4 (28.5–32.4)	25.9 (25.2–26.6)	1.17 (1.09–1.26)	1.08 (1.01–1.16)
Non-invasive ventilation				
No	25.2 (24.0–26.5)	14.7 (14.4–15.0)	1.72 (1.63–1.81)	1.06 (1.00–1.12)
Yes	39.6 (35.7–43.9)	33.3 (31.8–34.8)	1.19 (1.06–1.33)	0.98 (0.88–1.10)
Surgery performed, any type				
No	36.0 (33.9–38.1)	21.2 (20.6–21.7)	1.70 (1.59–1.81)	0.99 (0.93–1.06)
Yes	20.7 (19.3–22.2)	12.9 (12.5–13.3)	1.60 (1.49–1.73)	1.03 (0.95–1.12)
Cardiac surgery performed				
No	31.9 (30.5–33.3)	17.5 (17.1–17.8)	1.82 (1.74–1.92)	1.02 (0.97–1.08)
Yes	7.1 (5.7–8.9)	6.3 (5.7–6.9)	1.13 (0.89–1.44)	0.80 (0.52–1.23)
Abdominal surgery performed				
No	26.2 (24.9–27.6)	15.8 (15.5–16.2)	1.65 (1.56–1.75)	1.05 (0.99–1.11)
Yes	29.6 (26.7 -32.7)	16.7 (16.2–17.7)	1.75 (1.56–1.95)	1.02 (0.91 -1.14)
Orthopaedic surgery performed				
No	26.5 (25.3–27.7)	16.1 (15.7–16.4)	1.65 (1.57–1.74)	1.05 (1.00–1.11)
Yes	33.3 (27.7–39.7)	15.9 (14.6–17.4)	2.09 (1.71–2.56)	1.03 (0.80–1.31)
Vascular surgery performed				
No	27.2 (25.9–28.4)	16.4 (16.0–16.7)	1.66 (1.58–1.75)	1.04 (0.99–1.10)
Yes	16.8 (12.0–23.1)	10.4 (9.3–11.6)	1.61 (1.14–2.28)	0.64 (0.31–1.33)
365-day mortality				
Overall	40.9 (40.0–42.3)	25.4 (25.0–25.8)	1.61 (1.55–1.67)	1.03 (1.00–1.07)
Age				
15–55 yr	19.9 (15.5–25.3)	9.8 (9.4–10.3)	2.02 (1.57–2.59)	1.34 (1.06–1.69)
55–71 yr	28.0 (25.8–30.3)	24.8 (24.2–25.5)	1.13 (1.04–1.23)	1.07 (0.98–1.16)
71–105 yr	48.6 (46.9–50.4)	43.1 (42.4–43.9)	1.13 (1.08–1.17)	1.03 (1.00–1.07)
Sex				
Female	45.6 (43.4–48.9)	25.9 (25.4–26.5)	1.76 (1.67–1.86)	1.07 (1.01–1.13)
Male	38.1 (36.4–39.8)	25.0 (24.5–25.5)	1.53 (1.45–1.60)	1.01 (0.96–1.05)
Prior myocardial infarction				
Not present	39.9 (38.4–41.5)	24.8 (24.4–25.2)	1.61 (1.55–1.68)	1.02 (0.98–1.07)
Present	44.7 (41.8–47.8)	31.2 (29.9–32.5)	1.43 (1.33–1.55)	1.07 (0.99–1.16)
Congestive heart failure				
Not present	37.1 (35.5–38.8)	24.2 (23.8–24.6)	1.53 (1.46–1.61)	1.04 (0.99–1.08)
Present	48.4 (46.0––50.7)	43.8 (42.1–45.6)	1.10 (1.04–1.18)	1.04 (0.98–1.10)
Mechanical ventilation				
No	38.8 (38.1–41.6)	21.1 (20.6–21.5)	1.89 (1.80–1.99)	1.00 (0.95–1.05)
Yes	42.4 (40.4–44.6)	33.3 (32.6–34.0)	1.27 (1.21–1.34)	1.09 (1.04–1.15)
Renal replacement therapy				
No	39.6 (38.3–41.0)	24.4 (24.1–24.8)	1.62 (1.56–1.68)	1.03 (0.99–1.06)
Yes	63.4 (59.5–71.2)	59.2 (56.6–61.7)	1.10 (1.00–1.22)	1.04 (0.94–1.15)
Treatment with inotropes or vasopressors				
No	46.6 (39.2–54.6)	28.7 (26.4–31.2)	1.62 (1.35–1.95)	1.00 (0.82–1.21)
Yes	52.3 (45.1–60.0)	42.9 (40.3–45.6)	1.22 (1.04–1.43)	0.96 (0.82–1.12)
Non-invasive ventilation				
No	47.6 (41.6–54.0)	32.2 (30.3–34.3)	1.48 (1.28–1.71)	0.99 (0.85–1.15)
Yes	55.3 (45.1–66.0)	48.6 (44.6–52.7)	1.14 (0.92–1.40)	0.96 (0.78–1.18)
Surgery performed, any type				
No	50.1 (47.9–52.3)	30.6 (30.0–31.3)	1.64 (1.56–1.72)	0.96 (0.92–1.01)
Yes	34.8 (33.1–36.5)	22.2 (21.7–22.6)	1.57 (1.49–1.66)	1.07 (1.02–1.13)
Cardiac surgery performed				
No	47.6 (46.1–49.2)	27.6 (27.2–28.1)	1.72 (1.66–1.79)	1.00 (0.97–1.04)
Yes	14.9 (12.9–17.3)	10.2 (9.5–10.9)	1.47 (1.25–1.73)	1.11 (0.88–1.40)
Abdominal surgery performed				
No	39.7 (38.3–41.2)	24.7 (24.3–25.1)	1.61 (1.55–1.68)	1.03 (0.99–1.07)
Yes	46.4 (43.2–49.7)	28.6 (27.7–29.5)	1.62 (1.50–1.75)	1.04 (0.97–1.12)
Orthopaedic surgery performed				
No	40.3 (38.9–41.7)	25.5 (25.1–25.9)	1.58 (1.52–1.64)	1.03 (0.99–1.07)
Yes	54.4 (48.2–60.9)	24.1 (22.5–25.8)	2.27 (1.98–2.59)	1.11 (0.97–1.27)
Vascular surgery performed				
No	41.2 (39.8–42.6)	25.8 (25.4–26.2)	1.60 (1.54–1.66)	1.02 (0.99–1.06)
Yes	33.0 (26.6–40.4)	18.0 (16.6–19.5)	1.83 (1.47–2.29)	1.18 (0.88 -1.57)

*AF* atrial fibrillation, *RR* relative risk

95 % confidence intervals are given in parentheses

^a^Reference group was non-AF patients within each stratum

^b^Adjusted for age and sex, comorbidities, and consultations and services provided by the general practitioner

At 1 year following ICU admission, the mortality was 40.9 % (95 % CI 40.0–42.3 %) in patients with pre-existing AF and 25.4 % (95 % CI 25.0–25.8 %) in patients without AF (Table 3). The corresponding adjusted RR was 1.03 (95 % CI 1.00–1.07). Similar to the 30-day results, pre-existing AF was associated with a pronounced increase in mortality among individuals aged 15–55 years (adjusted RR 1.34, 95 % CI 1.06–1.69), whereas this was not the case in the older patients. Among patients receiving mechanical ventilation during the ICU admission, mortality at 1 year following ICU admission was increased in patients with AF compared with non-AF patients (adjusted RR 1.09, 95 % CI 1.04–1.15).

In patients with pre-existing AF, pre-admission users of vitamin K antagonists had lower 30-day and 1-year mortality compared with non-users (adjusted RR 0.91, 95 % CI 0.82–1.00; adjusted RR 0.91, 95 % CI 0.85–0.97) (Table 4). Reduced 30-day mortality in patients with AF was also seen with user of non-dihydropyridine calcium-channel blockers, but the estimate was rather imprecise (adjusted RR 0.86, 95 % CI 0.71–1.03), and the effect did not persist at 1 year of follow-up. Patients with AF using amiodarone had higher 30-day and 1-year mortality than non-users (adjusted RR 1.15, 95 % CI 0.98–1.34; adjusted RR 1.12, 95 % CI 1.01–1.25). Among patients with pre-existing AF, use of statins, aspirin, beta-blockers, and digoxin did not seem to influence the mortality at 30 days or 1 year following ICU admission.

**Table 4:** Mortality at 30 days and 365 days following admission in 57,110 adult ICU patients with and without pre-admission use of different drugs, according to atrial fibrillation status

	Users, %	Non-users, %	Crude RR^a^	Adjusted RR^a,b^
30-day mortality				
Statins				
No AF	15.8 (15.1–16.5)	16.1 (15.8–16.5)	0.98 (0.93–1.03)	0.83 (0.79–0.88)
AF	23.6 (21.7–25.7)	28.4 (26.9–30.0)	0.83 (0.75–0.92)	0.93 (0.84–1.04)
Aspirin				
No AF	20.1 (19.3–20.8)	15.0 (14.7–15.4)	1.34 (1.28–1.40)	0.93 (0.88–0.97)
AF	28.2 (26.3–30.3)	25.9 (24.4–27.5)	1.09 (0.99–1.20)	0.99 (0.91–1.09)
Vitamin K antagonists				
No AF	23.3 (21.1–25.7)	15.9 (15.5–1.62)	1.47 (1.33–1.62)	1.07 (0.97–1.19)
AF	22.9 (21.3–24.6)	30.6 (28.9–32.4)	0.75 (0.68–0.82)	0.91 (0.82–1.00)
Beta-blockers				
No AF	17.2 (16.4–17.9)	15.8 (15.4–16.1)	1.09 (1.02–1.12)	0.91 (0.86–0.95)
AF	26.4 (24.8–28.1)	27.3 (25.5–29.2)	0.97 (0.88–1.06)	1.07 (0.98–1.18)
Non-dihydropyridine CCBs				
No AF	21.4 (18.9–24.3)	16.0 (15.6–16.3)	1.34 (1.18–1.52)	1.04 (0.92–1.17)
AF	24.2 (20.1–28.9)	27.0 (25.8–28.3)	0.90 (0.74–1.08)	0.86 (0.71–1.03)
Digoxin				
No AF	32.7 (29.6–36.2)	15.8 (15.5–16.1)	2.07 (1.87–2.30)	1.15 (1.03–1.28)
AF	29.5 (27.4–31.8)	25.5 (24.0–27.0)	1.16 (1.05–1.27)	0.97 (0.88–1.07)
Amiodarone				
No AF	25.2 (18.3–34.2)	16.0 (15.7–16.3)	1.58 (1.15–2.16)	1.08 (0.83–1.42)
AF	27.3 (23.0–32.2)	26.8 (25.5 -28.0)	1.02 (0.86–1.21)	1.15 (0.98 -1.34)
365-day mortality				
Statins				
No AF	24.8 (24.0–25.6)	25.6 (25.1–26.0)	0.97 (0.94–1.01)	0.82 (0.79–0.86)
AF	36.7 (34.5–39.1)	43.1 (41.4–44.8)	0.85 (0.79–0.92)	0.95 (0.88–1.02)
Aspirin				
No AF	31.0 (30.2–31.9)	23.9 (23.5–24.4)	1.30 (1.25–1.34)	0.93 (0.90–0.96)
AF	42.7 (40.6–44.9)	39.8 (38.1–41.5)	1.07 (1.00–1.15)	1.00 (0.94–1.06)
Vitamin K antagonists				
No AF	37.8 (35.3–40.5)	25.1 (24.7–25.5)	1.51 (1.41–1.62)	1.11 (1.03–1.19)
AF	35.4 (33.5–37.3)	46.3 (44.4–48.3)	0.76 (0.71–0.82)	0.91 (0.85–0.97)
Beta-blockers				
No AF	26.9 (26.0–27.7)	25.1 (24.6–25.5)	1.07 (1.03–1.11)	0.89 (0.86–0.93)
AF	39.4 (37.6–41.3)	42.7 (40.7–44.8)	0.92 (0.86–0.99)	1.00 (0.95–1.07)
Non-dihydropyridine CCBs				
No AF	33.3 (30.3–36.5)	25.3 (24.8–25.6)	1.32 (1.20–1.45)	1.02 (0.94–1.11)
AF	39.4 (34.6–44.7)	41.0 (39.6–42.4)	0.96 (0.84–1.10)	0.93 (0.82–1.05)
Digoxin				
No AF	50.1 (46.6–53.6)	25.0 (24.7–25.4)	2.00 (1.86–2.15)	1.16 (1.08–1.24)
AF	45.0 (42.6 -47.4)	38.9 (37.3–40.6)	1.15 (1.08–1.24)	1.01 (0.95–1.08)
Amiodarone				
No AF	44.4 (35.8–53.9)	25.4 (25.0–25.7)	1.75 (1.42–2.15)	1.18 (0.99–1.41)
AF	42.2 (37.3–47.4)	40.8 (39.4–42.2)	1.03 (0.91–1.17)	1.12 (1.01–1.25)

AF atrial fibrillation, *RR* relative risk, *CCBs* calcium channel blockers

95% confidence intervals are given in parentheses

^a^Reference group was non-users within each stratum

^b^Adjusted for age and sex, comorbidities, and consultations and services by the general practitioner

## Discussion

This study of 57,110 ICU patients provides novel insights into the role of pre-existing AF on the prognosis for patients admitted to the ICU. Without confounder adjustment, our findings show that patients with pre-existing AF are at substantially higher risk of ATE and death following ICU admission than are patients without pre-existing AF. However, much of the excess risk of ATE and most of the excess risk of death is conveyed by AF’s being part of a complex that includes advanced age and a high burden of cardiovascular comorbidity. Of note, AF per se appeared to increase mortality in younger ICU patients and in those treated with mechanical ventilation. In patients with pre-existing AF, pre-admission users of vitamin K antagonists seemed to have a lower risk of ATE and had reduced mortality.

Biological mechanisms behind these findings cannot be clarified by this observational study. AF is a well-established risk factor for development of thromboembolic events [7]. In the general population, the stroke risk associated with AF is dependent on coexisting diseases, as expressed by the CHA_2_DS_2_-VASc score [13, 23]. In our cohort, AF was associated with a modestly increased risk of ATE even after we adjusted for the risk factors included in the CHA_2_DS_2_-VASc score, indicating that patients with pre-existing AF are more vulnerable to the mechanisms leading to increased risk of ATE in critical illness than are non-AF patients. These mechanisms may include increased procoagulant activity induced by the inflammatory response [33]. Frailty following critical illness could also be an explanation of the increased long-term risk of ATE. Even though our results did not unambiguously demonstrate reduced risk of ATE with pre-admission use of vitamin K antagonists, patients with AF who were vitamin K antagonist users had lower mortality at both 30 days and 1 year following ICU admission, illustrating that protection from hypercoagulation could be beneficial for AF patients admitted to the ICU.

In two previous studies, researchers have reported in-ICU mortality ranging from 25 % to 27 % in patients with pre-existing AF, which is compatible with our 30-day estimate [34, 35]. The intent of the previous studies was not to describe the prognostic impact of pre-existing AF in ICU patients, however, and no RR estimates were reported. Our finding of no excess mortality with pre-existing AF contrasts with reports of increased mortality in ICU patients with new-onset AF. Perhaps new-onset AF during ICU admission reflects greater disease severity rather than being a cause of increased mortality per se. Alternatively, patients with pre-existing AF may have adapted to the condition—physiologically or by use of medications—and could therefore be less susceptible to complications than ICU patients with new-onset AF. In ICU patients younger than 55 years of age and in those treated with mechanical ventilation, we did find increased mortality with AF. Young ICU patients have little comorbidity, and their absolute mortality is comparably low. Thus, on a relative scale, presence versus absence of AF is likely to impact mortality more in this age group than in older, multimorbid ICU patients, in whom the effect of AF is often conjugated with effects of many other conditions. The 1.12-fold relative mortality increase with AF in patients treated with mechanical ventilation may be of clinical relevance, given the high absolute mortality risk in this patient group. Tachycardia and irregular heart rate may reduce cardiac filling in patients with AF, which could be further compromised by the positive intrathoracic pressure stemming from mechanical ventilation. Alternatively, mechanical ventilation may be mainly a marker of greater disease severity during which pre-existing AF could have an impact on mortality in patients with severe haemodynamic instability due to diminished cardiac performance caused by AF itself.

Pre-admission use of beta-blockers and statins has previously been associated with favourable prognosis in a broad population of ICU patients [22, 36]. We did not find a beneficial effect of either statins or beta-blockers in patients with AF, however. Of note, our estimates in non-AF patients were comparable with those previously reported [22, 36].

### Strengths and limitations

Major strengths of this study include its population-based design, that it was conducted within a uniform health care system, the use of prospectively and independently sampled data, and virtually complete follow-up, thereby reducing the risk of selection problems seen in studies based on selected clinics or patient groups. AF is reliably coded in the DNPR with a positive predictive value in the range 93–99 % [19, 37, 38].

The positive predictive value for ischaemic stroke is 88–100 %. Of patients coded with unspecified stroke, 57–70 % have ischaemic stroke [39, 40]. As previously noted, we were unable to assess exact temporal relationships between diseases diagnosed during the same admission, and some cases of ATE may have been present at ICU admission, thereby overestimating the risk of ATE from AF following ICU admission. We have addressed this issue in our sensitivity analysis, but the data are likely to underestimate the true risk of ATE as we considered only ATE diagnosed after discharge from the entire hospital admission during which the patient was treated in the ICU.

The use of filled prescriptions as a proxy for use of pharmaceuticals implies that any non-adherence or discontinuation of a given drug before admission would introduce a bias towards the null. Likewise, as we did not have data on in-hospital treatments, discontinuation of a drug during admission could also lead to a downward bias. Indeed, as ICU patients often receive nutrition via a feeding tube, it is likely that many patients in this cohort have had their pre-admission oral medications paused or altered. Also, use of anticoagulants, such as low molecular weight heparins, during the ICU admission could influence the risks of ATE and death in patients with AF, but we lacked data to elaboration on this potential mechanism. Users of prophylactic medications may in general have greater awareness of own health and disease than non-users. The structure of the Danish health care system with tax-funded medical care and partial reimbursement of prescription drugs probably outbalances these effects to some extent, and previous research has shown that users of medications such as statins in Denmark in fact have an unhealthier lifestyle than non-users [41]. Still, we cannot rule out that our finding of a beneficial effect of pre-admission use of vitamin K antagonists is affected by a ‘healthy user/adherer bias’, or rather a ‘frail non-user bias’.

The detailed registries and our large dataset allowed us to control extensively for potential confounders, of which the disease categories included in the Charlson comorbidity index have been validated with a mean positive predictive value of 98 % [42]. Because there is a substantial overlap in the disease categories included in the Charlson comorbidity index and the CHA_2_DS_2_-VASc score, we also expect the coding of the latter to have a high validity, albeit residual confounding and confounding from unmeasured variables, such as diseases managed solely by the patient’s GP (which includes, e.g., uncomplicated hypertension and uncomplicated type 2 diabetes) and lifestyle factors, could still influence our estimates.

## Conclusions

Pre-existing AF is common in ICU patients and predicts increased risk of ATE and death. However, coexisting diseases and advanced age in patients with AF explain much of these findings. AF was an independent risk factor for ATE at 1 year following ICU admission and for death at 30 days and 1 year following admission in ICU patients treated with mechanical ventilation and in patients younger than 55 years of age. Pre-admission use of vitamin K antagonists improved the prognosis for patients with AF. At present, there are no standards for management of pre-existing AF in ICU patients, and this study highlights the urgent need for evidence-based clinical guidelines in this area.

## Key messages

- This population-based study shows that pre-existing AF is common in ICU patients and a marker of increased risk of ATE and death.
- The increased risk of ATE and death is largely explained by older age and more comorbidities in patients with AF.
- Pre-existing AF per se predicts increased mortality in ICU patients younger than 55 years of age and in ICU patients treated with mechanical ventilation.
- Pre-admission use of vitamin K antagonists improves the prognosis for ICU patients with pre-existing AF.

## Additional file

Additional file 1: Table S1. Sensitivity analysis—risk of arterial thromboembolism after ICU discharge at 30 days and 365 days following ICU admission in patients with and without atrial fibrillation. **Table S2.** Diagnostic codes used. **Table S3.** Procedural codes used. **Table S4.** Codes used for pharmaceuticals. (DOCX 20 kb)

## Abbreviations

AF: Atrial fibrillation
ATE: Arterial thromboembolism
CCB: Calcium channel blocker
CHA_2_DS_2_-VASc: Congestive heart failure, hypertension, age, diabetes, prior stroke/transient ischaemic attack/thromboembolism, vascular disease, and sex category
CI: Confidence interval
CRR: Cumulative risk ratio
DID: Danish Intensive Care Database
DNPR: Danish National Patient Register
GP: General practitioner
ICU: Intensive care unit
IQR: Interquartile range
RR: Relative risk
