# Supplementary material for: Pre-existing atrial fibrillation and risk of arterial thromboembolism and death in intensive care unit patients: a population-based cohort study
Source: Crit Care. 2015 Aug 19;19(1):299. doi: 10.1186/s13054-015-1007-5 (PMC4543470; doi:10.1186/s13054-015-1007-5)
Supplement: Additional file 1: Table S1. — Sensitivity analysis—risk of arterial thromboembolism after ICU discharge at 30 days and 365 days following ICU admission in patients with and without atrial fibrillation. Table S2. Diagnostic codes used. Table S3. Procedural codes used. Table S4. Codes used for pharmaceuticals. (DOCX 20 kb) [file 13054_2015_1007_MOESM1_ESM.docx]

Jacob Gamst, Christian Fynbo Christiansen, Bodil Steen Rasmussen, Lars Hvilsted Rasmussen, Reimar Wernich Thomsen: **Pre-existing atrial fibrillation and risk of arterial thromboembolism and death in intensive care unit patients: a population based cohort study**

**—Supplemental material—**

Contents

- Appendix Table 1: Sensitivity analysis—Risk of arterial thromboembolism after ICU discharge at 30 days and 365 days following ICU admission in patients with and without atrial fibrillation
- Appendix Table 2: Used diagnostic codes
- Appendix Table 3: Used procedural codes
- Appendix Table 4: Used codes for pharmaceuticals

Additional file 1: Table S1: Sensitivity analysis—Risk of arterial thromboembolism after ICU discharge at 30 days and 365 days following ICU admission in patients with and without atrial fibrillation

|  | **30-day risk** | | **365-day risk** | |
| --- | --- | --- | --- | --- |
|  | **No AF** | **AF** | **No AF** | **AF** |
| **Overall** |  |  |  |  |
| Cumulative risk, % | 0.2 (0.2 – 0.3) | 0.3 (0.1 – 0.5) | 1.0 (0.9 – 1.1) | 1.7 (1.3 – 2.0) |
| Cumulative risk ratio |  |  |  |  |
| Crude | 1 (ref) | 1.52 (0.91 – 2.53) | 1 (ref) | 1.69 (1.35 – 2.13) |
| Adjusted^a^ | 1 (ref) | 0.94 (0.35 – 2.52) | 1 (ref) | 1.24 (0.92 – 1.67) |

95% confidence intervals are given in parentheses

AF: Atrial fibrillation

^a^: adjusted for the risk factors included in the CHA_2_DS_2_-VASc-score, i.e.: congestive heart failure, hypertension, age, diabetes, prior stroke, vascular diseases and sex category

Additional file 1: Table S2: Used diagnostic codes

| **Disease category** | **Diagnostic codes^a^** |
| --- | --- |
| Atrial fibrillation: | ICD-10: I48  ICD-8: 427.93, 427.94 |
| Arterial thromboembolism (i.e. non-hemorrhage stroke or thromboembolism in arteries of the extremities, mesenteric arteries, or unspecified arteries): | ICD-10: I63 ex I63.6, I64, I74.2 – 74.4, I74.8 – 74.9 K55.0  ICD-8: 432-434, 436. 444.20 – 444.99, 445 |
| Myocardial infarction | ICD-10: I21; I22; I23  ICD-8: 410 |
| Congestive heart failure | ICD-10: I50; I11.0; I13.0; I13.2  ICD-8: 427.09; 427.10; 427.11; 427.19; 428.99; 782.49 |
| Peripheral artery disease | ICD-10: I70; I71; I72; I73; I74; I77  ICD-8: 440; 441; 442; 443; 444; 445 |
| Cerebrovascular disease | ICD-10: I60–I69; G45; G46  ICD-8: 430–438 |
| Dementia | ICD-10: F00–F03; F05.1; G30  ICD-8: 290.09–290.19; 293.09 |
| Chronic pulmonary disease | ICD-10: J40–J47; J60–J67; J68.4; J70.1; J70.3; J84.1; J92.0; J96.1; J98.2; J98.3  ICD-8: 490–493; 515–518 |
| Connective tissue disease | ICD-10: M05; M06; M08; M09; M30; M31; M32; M33; M34; M35; M36; D86  ICD-8: 712; 716; 734; 446; 135.99 |
| Ulcer disease | ICD-10: K22.1; K25–K28  ICD-8: 530.91; 530.98; 531–534 |
| Mild liver disease | ICD-10: B18; K70.0–K70.3; K70.9; K71; K73; K74; K76.0  ICD-8: 571; 573.01; 573.04 |
| Diabetes types 1 and 2 | ICD-10: E10.0, E10.1; E10.9; E11.0; E11.1; E11.9  ICD-8: 249.00; 249.06; 249.07; 249.09; 250.00; 250.06; 250.07; 250.09 |
| Hemiplegia | ICD-10: G81; G82  ICD-8: 344 |
| Moderate to severe renal disease | ICD-10: I12; I13; N00–N05; N07; N11; N14; N17–N19; Q61  ICD-8: 403; 404; 580–583; 584; 590.09; 593.19; 753.10–753.19; 792 |
| Diabetes with end-organ failure | ICD-10: E10.2–E10.8; E11.2–E11.8  ICD-8: 249.01–249.05; 249.08; 250.01–250.05; 250.08 |
| Any tumor | ICD-10: C00–C75  ICD-8: 140–194 |
| Leukemia | ICD-10: C91–C95  ICD-8: 204–207 |
| Lymphoma | ICD-10: C81–C85; C88; C90; C96  ICD-8: 200–203; 275.59 |
| Moderate to severe liver disease | ICD-10: B15.0; B16.0; B16.2; B19.0; K70.4; K72; K76.6; I85  ICD-8: 070.00; 070.02; 070.04; 070.06; 070.08; 573.00; 456.00–456.09 |
| Metastatic solid tumor | ICD-10: C76–C80  ICD-8: 195–198; 199 |
| AIDS | ICD-10: B21-B24  ICD-8: 079.83 |
| Valvular heart disease | ICD-10: I34, I35, I05, I06, I07, I08, I091, I098, I099, I36, I37, I38, I39  ICD-8: 394, 395, 396, 397, 398, 424 |
| Hypertension | ICD-10: I10 - I15  ICD-8: 400.09 – 404.99 |
| Transient cerebral ischemic attack | ICD-10: G45.0-2 G45.4-9  ICD-8: 435.09-99 |
| Alcoholism | ICD-10: F10.03, F10.4, G312, G621, G721, I 426, K292, K860, K70, R780,T51, Z714, Z721  ICD-8:291.09-99, 303.09-303.29, 303.91-303.99, 571.09-10 |
| Obesity | ICD-10: E65-E68, Z72.3  ICD-8: 277.99 |
| Stroke | ICD-10: I60 – I62 I63, I64, I69  ICD-8: 430–431 432-434, 436 |

^a^: Diagnostic codes in the Danish National Patient Registry are assigned by the treating physicians according to the 8^th^ revision of the *International Classification of Disease* (ICD-8) until 1993 and by the 10^th^ revision (ICD-10) since 1994. For a further description of the *International Classification of Diseases*, please refer to: http://www.who.int/classifications/icd

Additional file 1: Table S3: Used procedural codes

| **Surgical procedure** | **NOMESCO code^a^** |
| --- | --- |
| Surgery, any type | KA–KX |
| Cardiac surgery | KF |
| Abdominal surgery | KJ |
| Orthopaedic surgery | KN |
| Vascular surgery | KP |

^a^: Procedural codes in the Danish National Patient Register are assigned according to the *Nordic Medico-Statistical Committee’s (NOMESCO) Classification of Surgical Procedures.* For a further description of the *NOMESCO Classification of Surgical Procedures*, please refer to: http://www.nowbase.org/~/media/Projekt%20sites/Nowbase/Publikationer/NCSP/NCSP%201_16.ashx

Additional file 1: Table S4: Used codes for pharmaceuticals

| **Drug category** | **ATC classification code^a^** |
| --- | --- |
| Statins | MC10AA01 + MC10AA03 + MC10AA05 + MC1AA07+ MC10AA0X |
| Aspirin | MB01AC06 |
| Vitamin K-antagonists | MB01AA |
| Beta blockers | MC07, not MC07AA07, not MC07AA57 |
| Calcium channel blockers | MC08DA + MC08DB |
| Digoxin | MC01AA05 |
| Amiodarone | MC01BD01 |

^a^: Pharmaceuticals are coded in the Danish National Prescription Registry according to the *Anatomical Therapeutic Chemical (ATC) Classification System*. For a further description of the *ATC Classification System*, please refer to: http://www.whocc.no/
